# Recurrent neural network modeling of multivariate time series and its application in temperature forecasting

**DOI:** 10.1371/journal.pone.0285713

**Published:** 2023-05-19

**Authors:** Edward Appau Nketiah, Li Chenlong, Jing Yingchuan, Simon Appah Aram

**Affiliations:** 1 Department of Statistics, Taiyuan University of Technology, College of Mathematics, Taiyuan University of Technology, Taiyuan, People’s Republic of China; 2 College of Safety and Emergency Management Engineering, Taiyuan University of Technology, Taiyuan, People’s Republic of China; Texas A&M University, UNITED STATES

## Abstract

Temperature forecasting plays an important role in human production and operational activities. Traditional temperature forecasting mainly relies on numerical forecasting models to operate, which takes a long time and has higher requirements for the computing power and storage capacity of computers. In order to reduce computation time and improve forecast accuracy, deep learning-based temperature forecasting has received more and more attention. Based on the atmospheric temperature, dew point temperature, relative humidity, air pressure, and cumulative wind speed data of five cities in China from 2010 to 2015 in the UCI database, multivariate time series atmospheric temperature forecast models based on recurrent neural networks (RNN) are established. Firstly, the temperature forecast modeling of five cities in China is established by RNN for five different model configurations; secondly, the neural network training process is controlled by using the Ridge Regularizer (L2) to avoid overfitting and underfitting; and finally, the Bayesian optimization method is used to adjust the hyper-parameters such as network nodes, regularization parameters, and batch size to obtain better model performance. The experimental results show that the atmospheric temperature prediction error based on LSTM RNN obtained a minimum error compared to using the base models, and these five models obtained are the best models for atmospheric temperature prediction in the corresponding cities. In addition, the feature selection method is applied to the established models, resulting in simplified models with higher prediction accuracy.

## 1. Introduction

Weather forecasting has a great impact on the lives of people and is very challenging in terms of accurately predicting atmospheric temperature [1]. The availability of large weather datasets has made it possible for patterns in weather data to be learned by using deep learning techniques instead of statistical models and or traditional numerical methods. It is proven that the use of deep learning methods or models in weather analysis and forecasting performs better compared to other statistical or numerical models [1,2]. The study of weather forecasting makes it possible to know the occurrences of certain events in time and avoid damages that could be caused by those events. The unforeseen occurrence of events as a result of weather changes can affect human lives, human activities as well as productivity in agriculture and other sectors [3].

Several studies have been conducted on weather forecasting using neural networks [6–10]. Research has been conducted to improve the global numeric model of the European Centre for Medium-Range *Weather Forecasts* (ECMWF) for forecast accuracy using a neural network based on machine learning for predicting parameters such as air temperature or precipitation [4]. Weather and climate prediction through the learning of atmospheric dynamics based on artificial intelligence (AI) have several applications. The application of artificial intelligence has the potential of being the bedrock of system modeling of future weather, and climate observation [5]. It tends to achieve good accuracy of prediction [6]. The use of deep neural networks to learn the full dynamics of climate data provides a good forecast for the model which is achieved based on the present information and the past weather forecasts with past errors [7].

In the early parts of 2019, The United Kingdom recorded extreme warm temperatures which had not been experienced in the past 122 years with average daily temperatures of 18.3°C [3]. During summer, high temperatures were frequently recorded across certain cities in China such as the South-Eastern Hebei, Central and Western Shandong, etc. while extreme daily high temperatures were recorded in some cities in Yunnan in mid-April-June which is considered the highest temperature ever recorded since 1961 with average temperatures around 40°C [8]. The prediction of weather and climate change has progressed in China over the years and efforts are being made to ensure that there is a massive improvement in weather forecasting [9].

Temperature forecasting is very important because it helps to give warnings that are essential to protect properties and avoid a drastic reduction in productivity. As a result of the impact of weather conditions on human life and other related activities, this study aims at building a model for predicting atmospheric temperature. The study seeks to apply a deep learning algorithm rather than using the traditional numerical methods to develop a model to forecast atmospheric temperature using RNN. Detecting patterns in weather data is a complex task that requires some level of sophistication that traditional statistical models or traditional numerical methods lack because of the complexity of the large datasets. The study makes use of five features, namely: atmospheric temperature, air pressure, cumulative wind speed, dew point temperature, and relative humidity. The study applies Bayesian optimization in tuning the hyper-parameters to obtain the best parameters that give the best model for temperature prediction for the five cities selected in this study. The feature selection method was also applied to the datasets to obtain the important features that would contribute to the prediction of atmospheric temperature. The study also aims to help improve and understand the importance of deep learning algorithms in temperature forecasts using RNN and LSTM.

## 2. Review of related works

The application of artificial neural networks (ANN) for atmospheric temperature prediction is specifically to study the short-term temperature within a certain locality. Research conducted by Hayati and Zahra [10] in their study found that the use of a multi-layer perceptron to model a short-term temperature forecasting system obtained a minimum forecasting error, exhibited good performance and high prediction accuracy, and can be considered an appropriate method to model the STTF (short-term temperature forecast) systems. The method obtained a better prediction of temperature and humidity compared with other methods such as the gradient boosting tree, random forest, and linear regression [11]. Radhika and Shashi [12] employed a support vector machine (SVM) in the prediction of maximum temperature. The results from their study showed that the non-linear regression approach is more appropriate to train SVM for this task than using a multi-layer perceptron with backpropagation.

The nature of weather data is non-linear, and as a result, studies have been conducted on weather forecasting using non-linear statistics. It is known that empirical schemes that incorporate non-linearity perform better than those that do not [13]. The study conducted by Abhishek et al. [14] aimed at the applicability of the ANN by developing reliable and effective non-linear predictive models for weather forecasting. The non-linear nature of weather data makes the use of ANN more suitable than using other traditional and machine learning techniques [15]. Research conducted by Paras et al. [16] on ANNs with backpropagation for supervised learning predicted some features of weather such as temperature (maximum and minimum) and humidity based on existing features. The prediction was based on the trained ANN and the results indicated that the model can make good predictions with high accuracy. The statistical indicators considered in the study were found to be good for identifying the hidden patterns in the data except for kurtosis and skewness. A study by Baboo and Shereef [17], using observational data of the weather for a specific period, revealed that the ANN with backpropagation performed better than the traditional numerical models. The dataset used in the study had a lot of features such as temperature, dew point, humidity, sea level pressure, visibility, wind, gust speed, and precipitation. The study used a neural network algorithm to predict temperature. The main network algorithm used in this study was the backpropagation neural network technique. An ANN has been used in predicting future weather conditions in Madhya Pradesh, Bhopal in India based on 5-year (2005–2010) actual data from the meteorological department [18].

The application of deep neural networks which include deep convolutional networks, and convolutional recurrent neural network [19–21] have been used in climate and weather forecasting which have been applied to atmospheric temperature forecasts. Kreuzer et al. [22] compared the performance of deep learning models using convolutional LSTM and Seasonal autoregressive integrated moving average (SARIMA) and found that the deep learning method, convolutional LSTM, outperforms all the other methods considered. Zhang and Dong [19] also investigated the prediction of temperature using convolution recurrent neural network (CRNN), the results indicate that the CRNN performs better than the benchmark models considered in the study. Fente and Singh [23] applied the LSTM technique to predict future weather and the neural network was trained for different combinations. The study made use of these parameters; temperature, precipitation, wind speed, pressure, dew point visibility, and humidity for predicting future weather.

The most recent research investigated the use of neural networks to learn the full dynamics of a strongly simplified general circulation model, providing a good forecast of the model state many days ahead as well as stable long-period climate time series. The deep neural networks could also be used to some extent on a complex and realistic model but only for forecasting several days and not for creating climate runs [7]. A study involving predicting air temperature using recurrent neural networks with long-short term memory (LSTM) was conducted by Rahayu et al. [24]. The study used interpolation for values that are not easily understood or recognized, train the data with RNN and LSTM and finally test the model. The research compared two optimization models: The Stochastic Gradient Descent (SGD) and the Adam Optimizer. The research concluded that the optimization model, quantity of data, and data sharing can affect the results. Ozbek et al. [25] investigated the application of deep learning methods for predicting atmospheric air temperature (AAT) using LSTM. Their findings showed that the LSTM can predict AAT with high accuracy for short-term prediction with data collected over a long period. Park et al. [26] proposed the prediction of temperature with the use of refined missing data which uses the LSTM model; the model helps restore missing data. The results obtained indicated that the LSTM model with refinement obtained minimum error compared with using deep neural network (DNN) based and LSTM models without refinement or linear interpolation.

In addition, Biradar et al. [27] investigated the use of K-medoids and the naïve Bayes method in weather forecasting with temperature, humidity, and wind as features considered in the study. The system emphasized making predictions for weather forecasts considering prior information available. The research concluded that decision trees and K-medoid clustering are the appropriate deep learning techniques for weather forecasting. Wu et al. [28] proposed the use of naïve Bayesian in weather forecasting through the application of machine learning. The results showed that the method is attainable and effective. Jaseena and Kovoor [29] presented a systematic review of distinct weather forecasting approaches using available datasets. The research illustrated a precise classification of weather forecasting models. The results from the study indicated that hybrid models and deep learning models have proven to be more reliable models for weather forecasting.

## 3. Methods

### 3.1 Recurrent neural network

The research makes use of RNN [30] to forecast the weather temperature based on the data available. RNNs are a family of neural networks which is mainly used for handling sequential data. The RNNs operate on a sequence with subvectors *x*(*T*) and time step index *t* ranging from 1 to *T*. Pragmatically, RNNs usually operate on the minibatch of such sequences, with a different sequence length *T* for each member of the minibatch. The recurrent network in a dynamic system is of the form $s^{t}=f(s^{\left(t-1\right)};\theta )$, where *s*^(*t*)^ is called the state of the system, and the system is recurrent because the definition of *S* at time *t* refers back to the same definition at the time *t*−1. The recurrent network with information on the input variable and the past information can be represented as, $s^{t}=f(s^{\left(t-1\right)},x^{\left(t\right)};\theta )$. This can also be expressed to indicate that the state is the hidden unit of the network, $h^{t}=f(h^{\left(t-1\right)},x^{\left(t\right)};\theta )$ [31]. The RNNs usually have optimization difficulty known as vanishing gradient which is alleviated by the LSTM which is adopted in this research. A multi-variate input of vectors *x*(*ij*) was used in the study with a timestep of 1 hour.

#### 3.1.1 Long Short-term Memory (LSTM)

**Fig 1:**
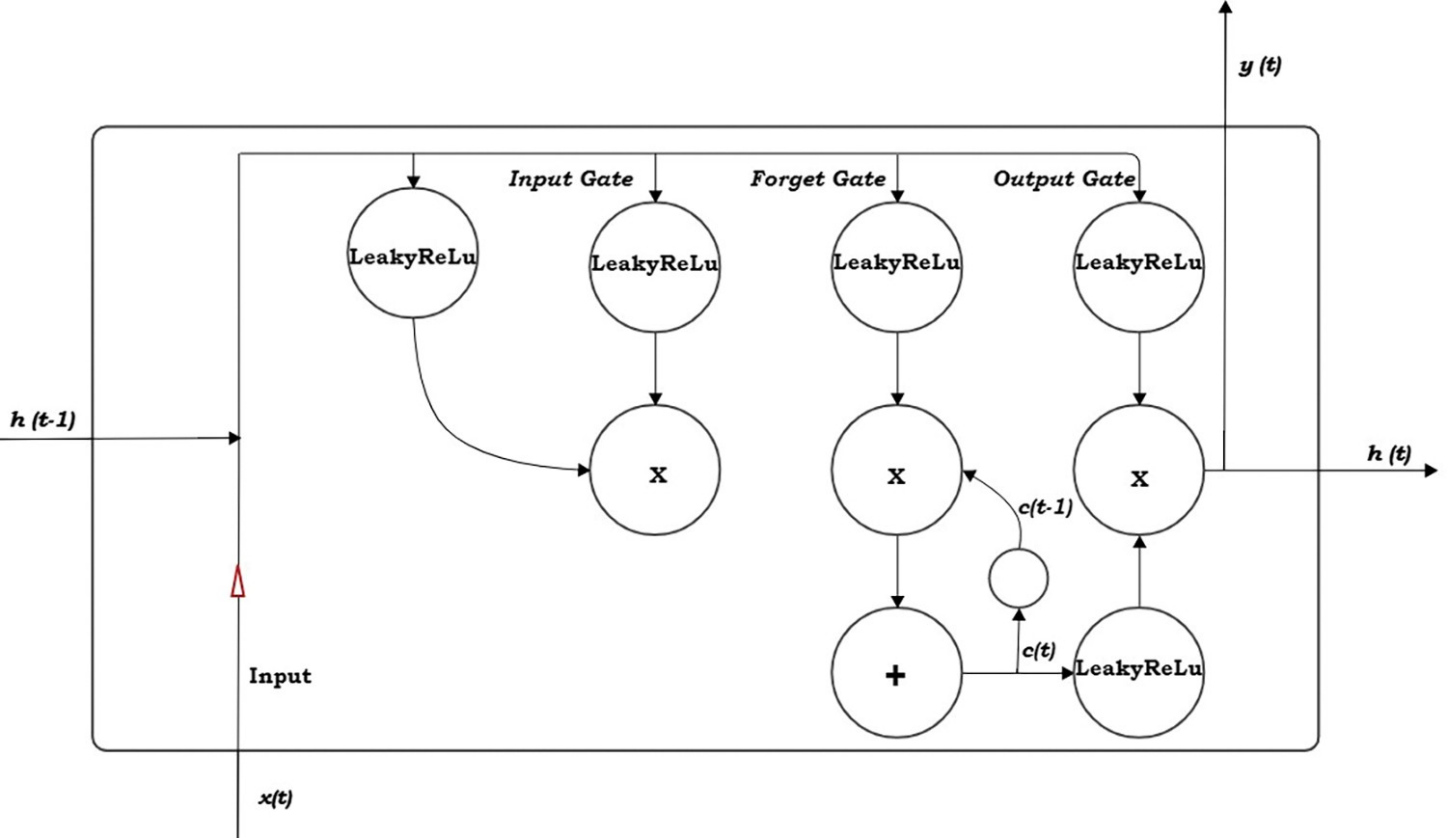
Structure of long-short term memory.

LSTM is used for modeling sequential data such as time-series data which is collected over time. The LSTM is made up of an input gate, an output gate, and forgets gate, as shown in Fig 1. The Elman and Jordan networks are termed a simple recurrent network, the complex architecture of the network is the LSTM.

This study makes use of the LeakyRelu activation function (Fig 1) because of its ability to solve the dying problem of the Relu activation function. The LeakyRelu activation function is max(0,*αz*,*z*), where *α* is the constant gradient. The appropriate value of *α* was chosen, which was set at 0.3.

The function is defined as:

$$f(z)=\{\begin{array}{c} z\;z>0\\ \alpha z\;z\leq 0 \end{array}$$ (1)

The derivative of the LeakyRelu activation function is given as $f^{\prime }(z)=\{\begin{array}{c} 1\;z>0\\ \alpha \;z<0 \end{array}$ The implementation of the backpropagation algorithm requires the cost function or error, the weighted input, and the activation function. The cost function is expressed as:

$$\mathrm{cost}\;\mathrm{function}=\frac{1}{2}{\sum \left(x_{n}-o_{n}\right)}^{2}$$ (2)

and the weighted input *z*, *z* = *xw*. The backpropagation algorithm requires many iterations as the weights get updated through the entire network [32–34]. The L2 regularizer was also employed in this study [35,36].

#### 3.1.2 RNN configuration

We developed an RNN, which comprises of 3-layers of LSTM cells. The configuration consists of the best-performed LSTM architecture for the 3-layers after the Bayesian optimization. The RNN configuration includes an L2 regularizer and LeakyReLU activation function at all layers of the network. We developed five models to predict the atmospheric temperature for the five selected cities.

### 3.2 Hyper-parameter optimization

The training of neural networks is a very tedious task. The best alternative to training a deep neural network is optimization. The use of optimization algorithms provides the best hyper-parameters such as the learning parameters, regularization parameters, etc., that perform well with the model architecture selected. The training of a deep neural network does not only depend on a large amount of data, choosing the optimal model hyper-parameters that can learn the algorithm is also a major factor in training a neural network.

There are different forms of optimization; we will make use of the Bayesian optimization with a Gaussian process (GP) prior. The Bayesian optimization can be considered as a process of minimizing an objective function *f*(*x*) over a bounded set of parameter values *x*, *x*∈ℝ^+^, the parameter values *x* are made up of the range of values for the hyper-parameters. The Bayesian optimization is well suited for our study since we have an unknown objective function *f*(*x*) to optimize, non-convex and very expensive to evaluate, in order to minimize the cost or risk involved and maximize the expected utility of the hyper-parameters. The Bayesian optimization makes use of the prior, and evidence (data) to obtain the posterior distribution over the space of functions [37]. The Bayesian optimization constructs a probabilistic model for *f*(*x*) and determines the next point of *x* to evaluate using an acquisition function selected. In this study, we selected the expected improvement (EI) with the GP and define a kernel for the process.

To implement the Bayesian optimization, we use the GPyOpt [38]. The objective function is approximated by a surrogate model, and the objective function *f*(*x*) is a black-box function. The execution of the GPyOpt iterates over a range of values for the hyper-parameters called bounds or domain and the best hyper-parameters are reported. This is used for further processing.

### 3.3 Data collection

The data was obtained from the UCI machine learning repository[39]. This data package contains hourly temperature data and other important features for 24 hours. It is made up of weather data on five cities in China namely: Beijing, Shanghai, Guangzhou, Chengdu, and Shenyang. According to Liang et al. [40], the data are collected from the airport of Beijing (40.072498,116.597504), Shanghai (31.143333,121.805275), Chengdu (30.66667, 104.06667), and Shenyang (41.637330784, 123.483498066); however, the weather data for Guangzhou is obtained from Central Meteorological Agency (CMA) site which is on the same side with the consulate. The datasets comprise atmospheric temperature, dew point temperature, relative humidity, air pressure, and cumulative wind speed.

#### 3.3.1 Data pre-processing and data analysis

The data were first normalized. This is the process of scaling data to fall within a smaller range. The data were normalized to fall within the range of 0 and 1. The normalized data was used since the input features have different measurements. Normalizing the data helps in speeding up the learning phase. This helps the data to converge faster than using the actual data values, the normalizing was done independently after splitting. The data is then reverted to its original state after the learning process. The data was split into 90% for training which includes 10% for validation and the remaining 10% for testing for the half-year. For the 1-year prediction, we used 80% for training (20% of the training set for validation) and the remaining 20% of the data for testing. There were few missing observations in the datasets for each city for the different variables considered in this study: Beijing (≤0.006), Guangzhou (≤0.00001), Shanghai (≤0.0005), Chengdu (≤0.01), and Shenyang (≤0.0132). We investigated with different methods, linear interpolation, backward filling, and forward filling to impute the missing observations, the best validation result for each of the five cities were used in the study.

#### 3.3.2 Training

The training of the neural network was done with a backpropagation algorithm. A learning rate that speeds up the training process and controls the speed of the network was chosen, which we set at a rate of 0.001. The LeakyReLU activation function was used with callbacks to help improve the model during the training process. The LeakyReLU is chosen because it attempts to fix the dying ReLU problem. The training process comprises 90% of the dataset, and 10% of the training dataset was used for validation. The inputs of the backpropagation network are atmospheric temperature (temp), dew point temperature, relative humidity, air pressure (press), and cumulative wind speed (wnd_spd).

#### 3.3.3 Prediction model based on RNN and LSTM

The dataset was modeled in such a way that the relationship between the current/future temperature and the past events of the dataset can be learned by the network. The LSTM accepts input of 3 tensors; with a timestep or lookback of 1, the data was modeled to learn from the previous events i.e. features used in the analysis, and make a prediction for the next hour since the data was collected hourly. This can be expressed as:

$$y_{t+1}=f(z_{t})$$ (11)

where *y*_*t*+1_ is the prediction of the target for the next hour and *f*(*z*_*t*_) is the function used to make predictions based on past observation.

We also make predictions for the next 3 days using information from time *t*−72 to *t*−1, the model is defined as:

$$f(z_{t-72},z_{t-71},z_{t-70},\ldots ,z_{t-1})=({\hat{y}}_{t},{\hat{y}}_{t+1},{\hat{y}}_{t+2},\ldots ,{\hat{y}}_{t+71})$$ (12)

where *z*_*t*−*i*_ is the input variables and ${\hat{y}}_{t+i}$ is the predicted temperature for the *i*th hour for the 3 days.

#### 3.3.4 Testing

The testing of the model was done on the remaining 10% of the dataset. The actual values and the predicted values were compared to determine the predictive ability of the model after obtaining the best hyper-parameters using Bayesian optimization.

### 3.4 Feature selection

**Fig 2:**
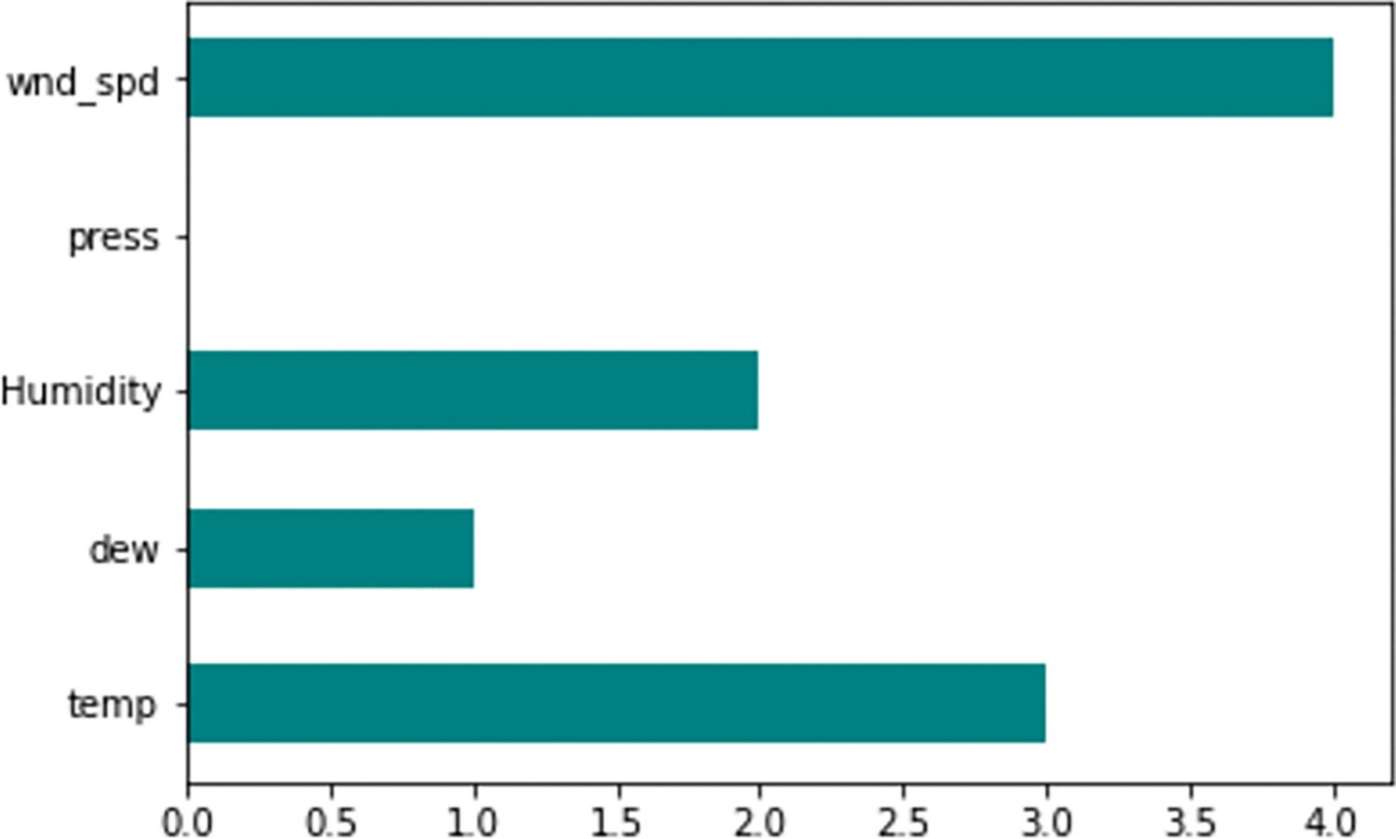
Feature importance of the variables.

The feature selection method was used to select the features that have an impact on the prediction of atmospheric temperature (Fig 2). The Fisher’s score indicated that wind speed, Humidity, and dew are the important features that contribute to the prediction of atmospheric temperature in this study. Moreover, we observed that there was a moderate correlation between dew and Humidity compared with the other variables. In addition, we also observed that there was a strong correlation between dew and the target variable (atmospheric temperature (°C)). We, therefore, retained dew and removed Humidity from the model. The input variables after feature selection were atmospheric temperature (temp), dew point temperature (dew), and cumulative wind speed (wnd_spd).

### 3.5 Evaluation criterion

#### 3.5.1 Mean Absolute Error (MAE)

The mean absolute error is a measure of the average of the difference between the actual observations and the predicted observations. The mean absolute error can be presented mathematically as:

$$\mathrm{MAE}=\frac{1}{n}\sum_{i=1}^{n}\left|y_{i}-{\hat{y}}_{i}\right|$$ (13)

#### 3.5.2 Mean squared error

The mean squared error measures the amount of error in a model. It assesses the average difference between the actual and the predicted observation. This can be mathematically expressed as:

$$\mathrm{MSE}=\frac{1}{n}\sum_{i=1}^{n}{\left(y_{i}-{\hat{y}}_{i}\right)}^{2}$$ (14)

#### 3.5.3 Binary accuracy measure

The binary accuracy measure is a metric that measures the frequency with which the predicted observations match the actual observations. It measures how often the developed model gets the prediction correct. It can be defined as:

$$\mathrm{Accuracy}=\frac{\mathrm{Number}\;\mathrm{of}\;\mathrm{correct}\;\mathrm{predictions}}{\mathrm{Total}\;\mathrm{number}\;\mathrm{of}\;\mathrm{predictions}}$$ (15)

We defined the accuracy measure as:

$$\mathrm{Accuracy}=\frac{1}{n}\sum_{i=1}^{n}I(|1-\frac{{\hat{y}}_{i}}{y_{i}}|<0.05)$$

where *n* is the length of the dataset, $\hat{y}$ is the predicted temperature, and *y* is the actual temperature.

#### 3.5.4 Coefficient of determination (*R*^2^)

The coefficient of determination measures the proportion of variability in the predicted variable explained by the independent variable, the value ranges from 0 to 1. It can be expressed mathematically as:

$$R^{2}=1-(\frac{\sum_{i=1}^{n}{\left(\hat{y}-y_{i}\right)}^{2}}{\sum_{i=1}^{n}{\left(\bar{y}-y_{i}\right)}^{2}})$$ (16)

## 4. Results and discussion

We found that the five datasets from the five cities in China performed differently with different model architectures. The dataset for each city was trained, validated, and tested based on the best hyper-parameters that were chosen after performing the Bayesian optimization using the GPyOpt. The hyper-parameters obtained best predict the temperature for each of the selected cities. We used the Adam Optimizer [41] with early stopping and standard LSTM cells.

### 4.1 Datasets

We used five different datasets from five different cities in China: Beijing, Shanghai, Guangzhou, Chengdu, and Shenyang for the study. The dataset comprised hourly atmospheric temperature, dew point temperature, relative humidity, air pressure, and cumulative wind speed data from the five cities for 24 hours each from 2010 to 2015. There were a few missing observations in the datasets which we imputed with the nearest observation, we found that the number of missing observations would not have any significant influence on the data. Through the Bayesian optimization of hyper-parameters, five model configurations for the five cities were chosen that best predict the atmospheric temperature with less error. The mean absolute error (MAE) and the mean squared error (MSE) were used to evaluate the model during training and predictions. Table 1 contains the best model configuration selected for each of the five cities with other accuracy comparison measures. The table also shows the base models: ordinary least squares regression (OLS) and lasso regression (LASSO). Table 2 also presents the results for the selected models after training and comparing the prediction using at least a 1-year dataset with other accuracy comparison measures.

**Table 1:** Model configurations and comparison measures of half-year.

			Evaluation Criterion			
**City**	**Models**	**R-Squared**	**MAE**	**MSE**		
Beijing	OLS	Training (0.9666)	Training (1.6573)	Training (4.9986)		
		Test (0.9613)	Test (1.7612)	Test (5.5801)		
	LASSO	Training (0.9652)	Training (1.6536)	Training (5.0977)		
		Test (0.9647)	Test (1.6540)	Test (5.1651)		
Guangzhou	OLS	Training (0.8053)	Training (2.3861)	Training (9.1562)		
		Test (0.9913)	Test (2.1693)	Test (7.3752)		
	LASSO	Training (0.8076)	Training (2.3446)	Training (8.8578)		
		Test (0.8077)	Test (2.341)	Test (8.8226)		
Shanghai	OLS	Training (0.9888)	Training (0.5812)	Training (1.0109)		
		Test (0.9904)	Test (0.5636)	Test (0.6808)		
	LASSO	Training (0.9897)	Training (0.6013)	Training (0.8896)		
		Test (0.9893)	Test (0.6046)	Test (0.9317)		
Chengdu	OLS	Training (0.985)	Training (0.6493)	Training (0.9931)		
		Test (0.9757)	Test (0.6969)	Test (0.3588)		
	LASSO	Training (0.9842)	Training (0.5885)	Training (1.0077)		
		Test (0.9853)	Test (0.5827)	Test (0.9442)		
Shenyang	OLS	Training (0.6614)	Training (2.1025)	Training (66.4326)		
		Test (0.98467)	Test (1.2161)	Test (2.7613)		
	LASSO	Training (0.9865)	Training (1.0561)	Training (2.6006)		
		Test (0.9883)	Test (1.0427)	Test (2.2583)		
	**Models**	**Configuration**	**MAE**	**MSE**	**Accuracy**	**Loss**
Beijing	RNN + LSTM	3 layers, activation function: LeakyRelu (alpha = 0.3),	Training (0.086)	Training (0.0163)	Train score (91.73%)	0.00028
		L2 Regularizer (0.0001),	Val (0.102)	Val (0.0166)	Val score (81.79%)	
		Adam Optimizer (lr = 0.001), patience = 10, LSTM(256,64,64)	Test (0.065)	Test (0.0102)	Test score (95.13%)	
Guangzhou	RNN + LSTM	3 layers, activation function: LeakyRelu (alpha = 0.3),	Training (0.122)	Training (0.0290)	Train score (99.25%)	0.0015
		L2 Regularizer (0.001),	Val (0.092)	Val (0.0152)	Val score (99.39%)	
		Adam Optimizer (lr = 0.001), patience = 10, LSTM(512,256,128)	Test (0.125)	Test (0.0237)	Test score (99.90%)	
Shanghai	RNN + LSTM	3 layers, activation function: LeakyRelu (alpha = 0.3),	Training (0.127)	Training (0.0276)	Train score (99.95%)	0.01149
		L2 Regularizer (0.0001),	Val (0.102)	Val (0.02002)	Val score (100%)	
		Adam Optimizer (lr = 0.001), patience = 10, LSTM(512,256,256)	Test (0.130)	Test (0.0267)	Test score (99.94%)	
Chengdu	RNN + LSTM	3 layers, activation function: LeakyRelu (alpha = 0.3),	Training (0.170)	Training (0.0482)	Train score (99.26%)	0.0059
		L2 Regularizer (0.01),	Val (0.117)	Val (0.0218)	Val Score (99.56%)	
		Adam Optimizer (lr = 0.001), patience = 10, LSTM(1024,256,128)	Test (0.195)	Test (0.0567)	Test score (99.87%)	
Shenyang	RNN + LSTM	3 layers, activation function: LeakyRelu (alpha = 0.3),	Training(0.211)	Training (0.0798)	Train score (84.07%)	0.0027
		L2 Regularizer (0.01),	Val (0.277)	Val (0.1358)	Val score (72.32%)	
		Adam Optimizer (lr = 0.001), patience = 10, LSTM(256,64,64)	Test (0.211)	Test (0.0987)	Test score (83.02%)	

**Table 2:** Model configurations and comparison measures of 1-year.

		Configuration	MAE	MSE	Accuracy	Loss
Beijing	RNN + LSTM	3 layers, activation function: LeakyRelu (alpha = 0.3),	Training (0.180)	Training (0.0572)	Train score (86.28%)	0.00054
		L2 Regularizer (0.0001),	Val (0.179)	Val (0.0567)	Val score (85.16%)	
		Adam Optimizer (lr = 0.001), patience = 10, LSTM(256,64,64)	Test (0.163)	Test (0.0455)	Test score (80.88%)	
Guangzhou	RNN + LSTM	3 layers, activation function: LeakyRelu (alpha = 0.3),	Training (0.117)	Training (0.0250)	Train score (99.43%)	0.1267
		L2 Regularizer (0.1),	Val (0.126)	Val (0.3263)	Val score (99.07%)	
		Adam Optimizer (lr = 0.001), patience = 10, LSTM(512,256,64)	Test (0.107)	Test (0.0228)	Test score (99.90%)	
Shanghai	RNN + LSTM	3 layers, activation function: LeakyRelu (alpha = 0.3),	Training (0.140)	Training (0.0360)	Train score (99.78%)	0.0004
		L2 Regularizer (0.0001),	Val (0.128)	Val (0.0239)	Val score (100%)	
		Adam Optimizer (lr = 0.001), patience = 10, LSTM(512,256,256)	Test (0.119)	Test (0.0218)	Test score (100%)	
Chengdu	RNN + LSTM	3 layers, activation function: LeakyRelu (alpha = 0.3),	Training (0.172)	Training (0.0438)	Train score (99.31%)	0.0069
		L2 Regularizer (0.01),	Val (0.158)	Val (0.0358)	Val Score (99.38%)	
		Adam Optimizer (lr = 0.001), patience = 10, LSTM(1024,256,128)	Test (0.152)	Test (0.0341)	Test score (99.70%)	
Shenyang	RNN + LSTM	3 layers, activation function: LeakyRelu (alpha = 0.3),	Training(0.156)	Training (0.0449)	Train score (90.82%)	0.0031
		L2 Regularizer (0.01),	Val (0.153)	Val (0.0694)	Val score (89.99%)	
		Adam Optimizer (lr = 0.001), patience = 10, LSTM(256,64,64)	Test (0.150)	Test (0.0513)	Test score (88.94%)	

### 4.2 Short-term prediction

We trained each of the datasets with the training dataset and validated the model after training with the validation dataset. For each dataset, we predicted the training set, the validation set, and the testing set. The results were used together with the actual training set, validation set, and testing set to compute the MAE and the MSE for the evaluation criterion in Table 1.

The binary accuracy used to compare the actual values and the predicted values indicated that the performance was very high for all five cities based on the model architecture selected. In Table 1, we observed a high prediction accuracy (72.32% - 100%) for all five selected cities for the training set, validation set, and test set based on the configuration selected through the use of Bayesian optimization in selecting the best hyper-parameters for each city. Beijing achieved a high prediction accuracy for training (91.73%), validation set (81.79%), and test prediction (95.13%). For the remaining cities, Guangzhou obtained a fairly high prediction accuracy: train prediction (99.25%), validation prediction (99.39%), and test prediction (99.90%). Shenyang achieved a high train prediction (84.07%), validation prediction (72.32%), and test prediction (83.02%), Shanghai obtained a nearly perfect prediction accuracy for the train set (99.95%) and test set (99.94%), and a perfect prediction for the validation set (100%). Chengdu also obtained a high prediction accuracy for the train set (99.26%), and perfect prediction accuracy for both the validation set (99.56%) and test set (99.87%). We employed the L2 regularizer in the study to help prevent overfitting or underfitting during training. The RNNs architectures can efficiently learn the pattern of the input data and make predictions based on the input learned.

**Fig 3:**
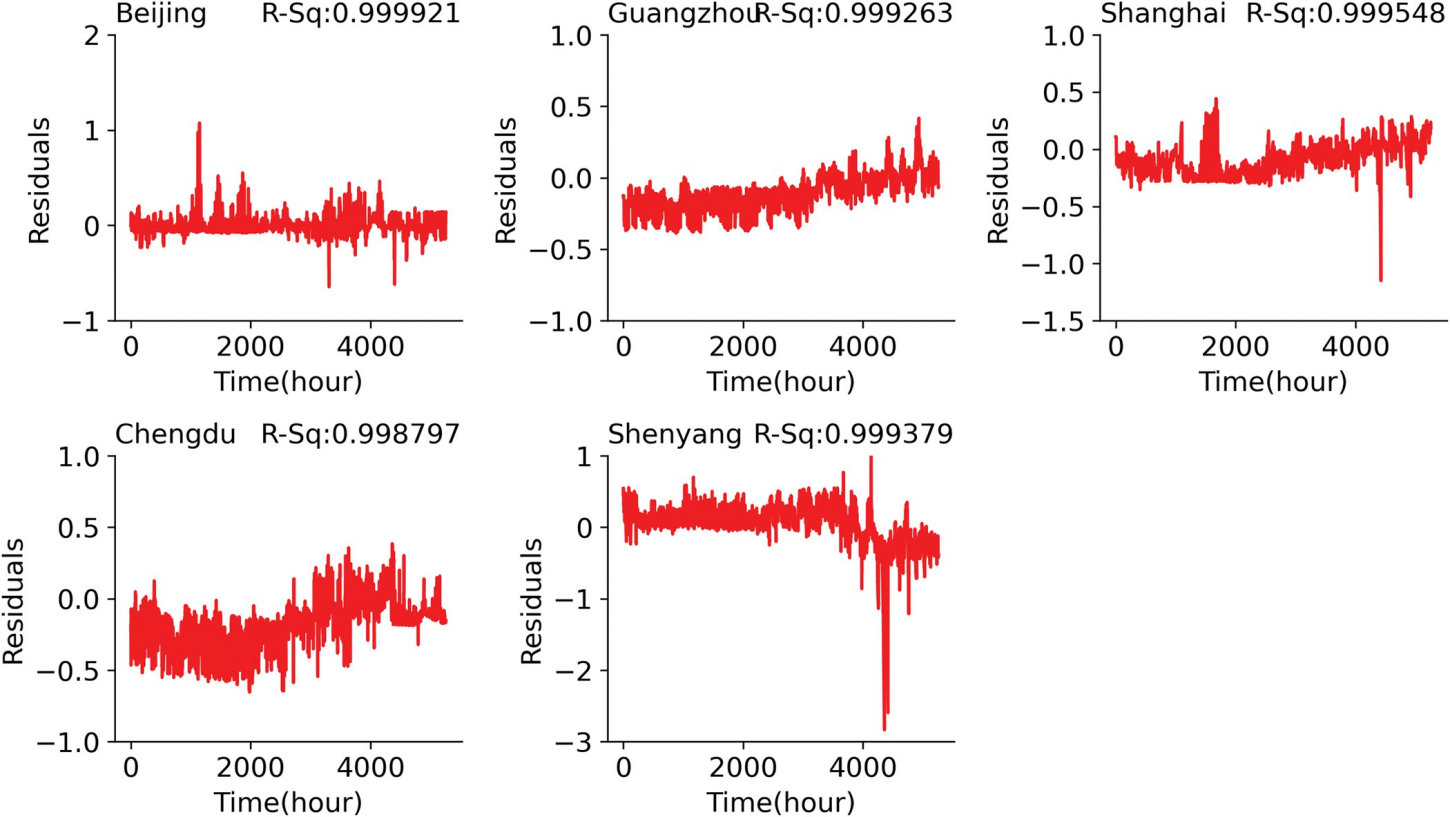
Residual plot of the predicted and actual temperature test datasets for the half-year dataset.

The plot in Fig 3 indicates that models achieved high performance in prediction with high *R*^2^ values in all the plots of the actual atmospheric temperature and the predicted atmospheric temperature using the short-term half-year prediction for all the cities.

**Fig 4:**
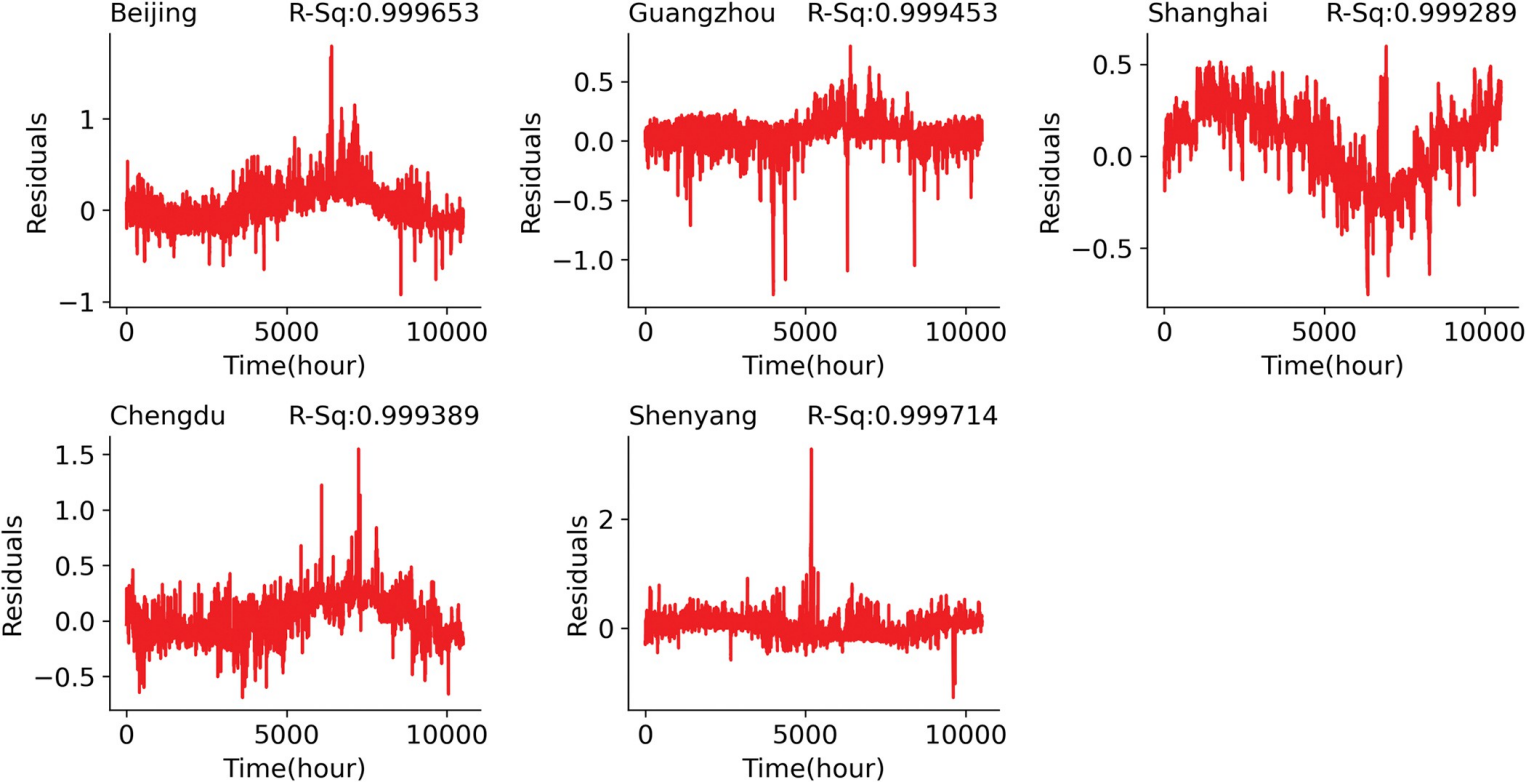
Residual plot of the predicted and actual temperature test datasets for the 1-year short-term prediction.

The short-term prediction using at least a one-year dataset obtained an equivalently high performance as using the half-year dataset for prediction. The 1-year short-term prediction also obtained minimal errors: Beijing (MSE: 0.0455–0.0572, MAE: 0.163–0.180), Guangzhou (MSE: 0.0228–0.3263, MAE: 0.107–0.126), Shanghai (MSE: 0.0218–0.0360, MAE: 0.119–0.140), Chengdu (MSE: 0.0341–0.0438, MAE: 0.152–0.172) and Shenyang (MSE: 0.044–0.0694, MAE: 0.150–0.156). The 1-year short-term prediction also achieved high prediction accuracy (80.88%– 100%). The comparison plot of the actual and the predicted values for the 1-year short-term prediction can be seen in Fig 4.

**Fig 5:**
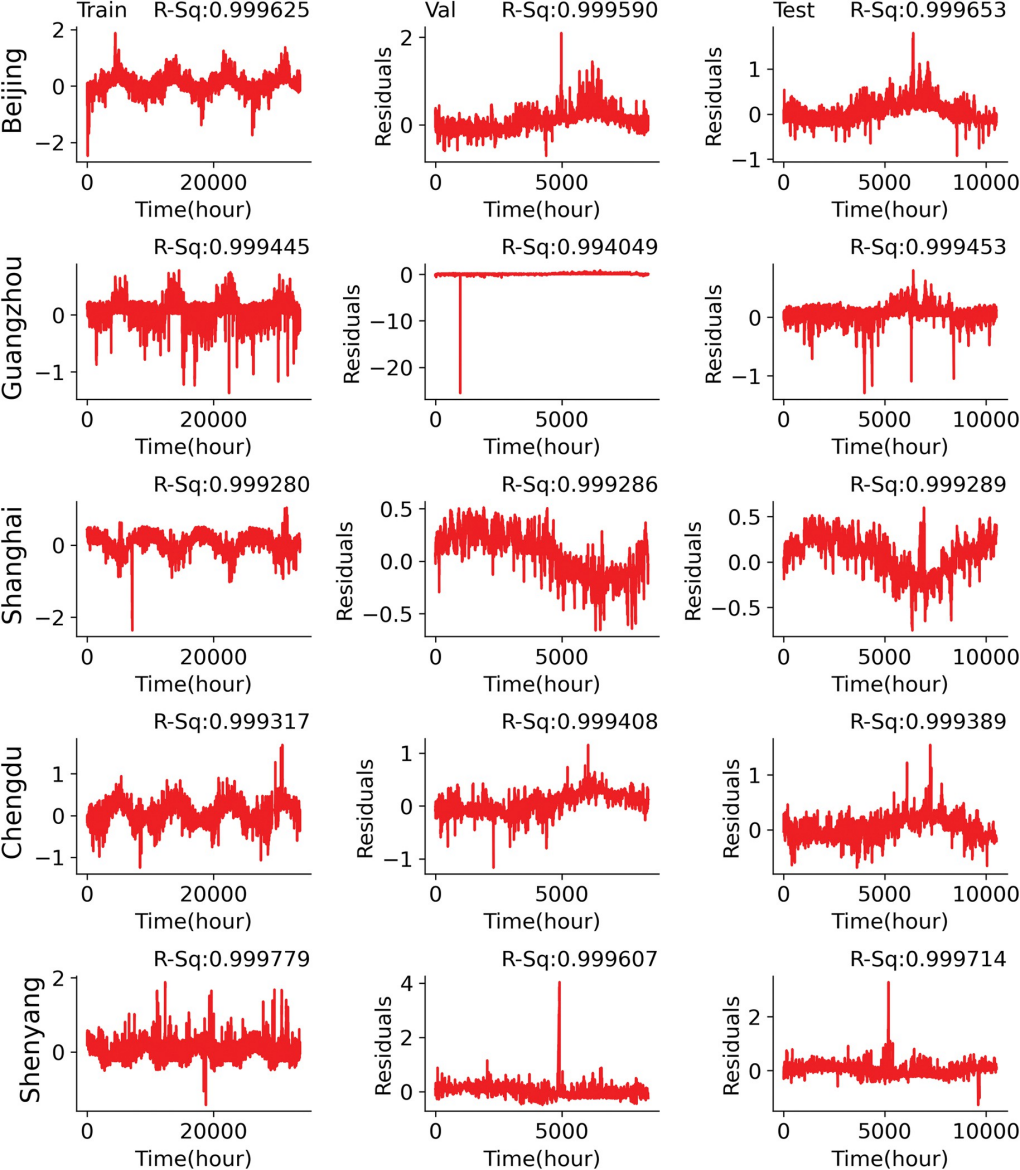
Scatterplot of the actual data versus train prediction, validation prediction, and test prediction for the 1-year dataset.

The selected model architectures obtained after using Bayesian optimization can be considered the best predictor of temperature for the selected cities. Fig 5 presents the residual plot of the actual dataset for 2010–2015; the training prediction plot, the validation prediction plot, and the test prediction plot for the five selected cities. The plot indicates that models achieved high performance in prediction with high *R*^2^ values in all the plots of the actual atmospheric temperature and the predicted atmospheric temperature for the selected cities.

### 4.3 Long-term prediction

**Fig 6:**
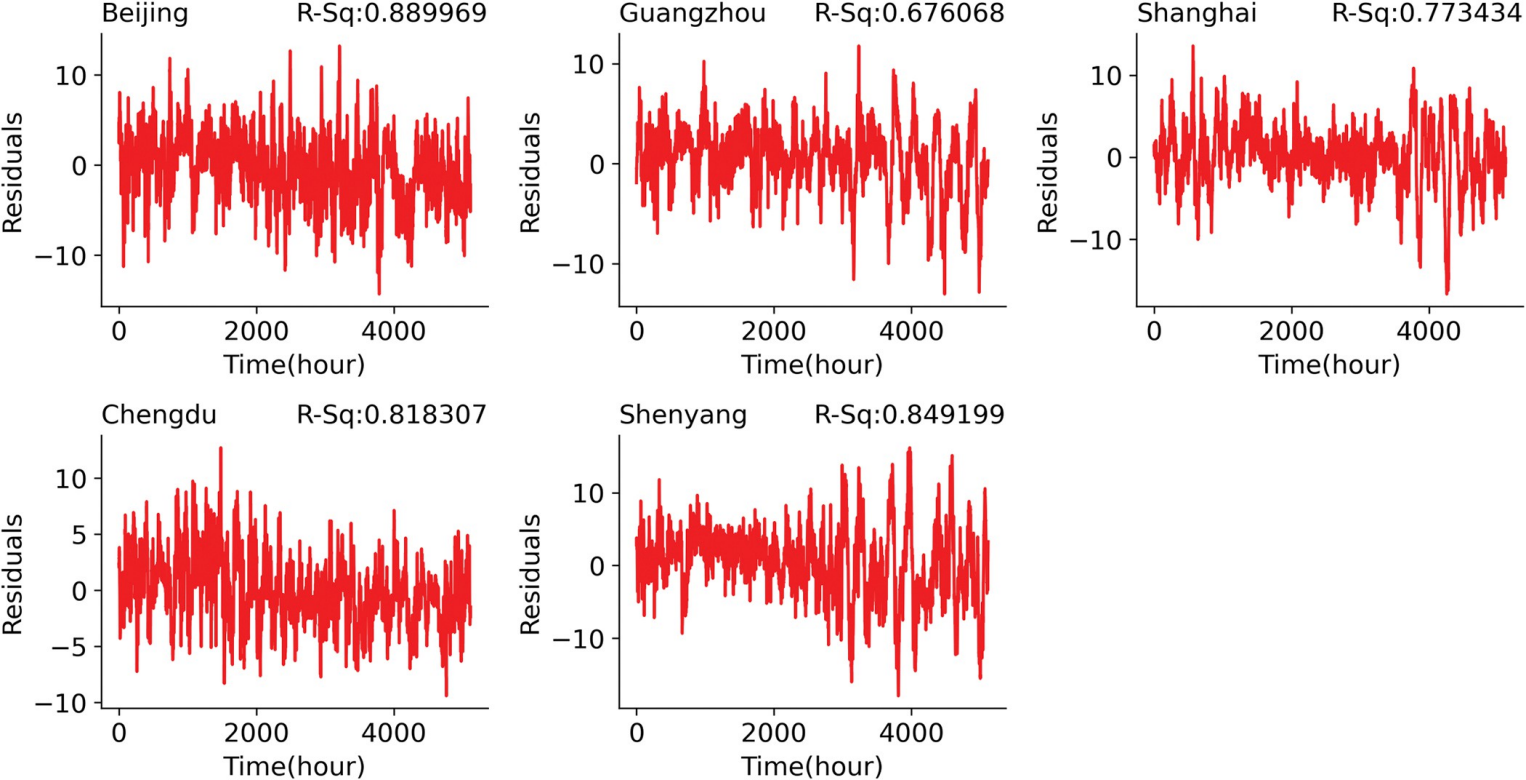
Residual plot of the actual test dataset and predicted temperature (72nd-hour predictions).

**Fig 7:**
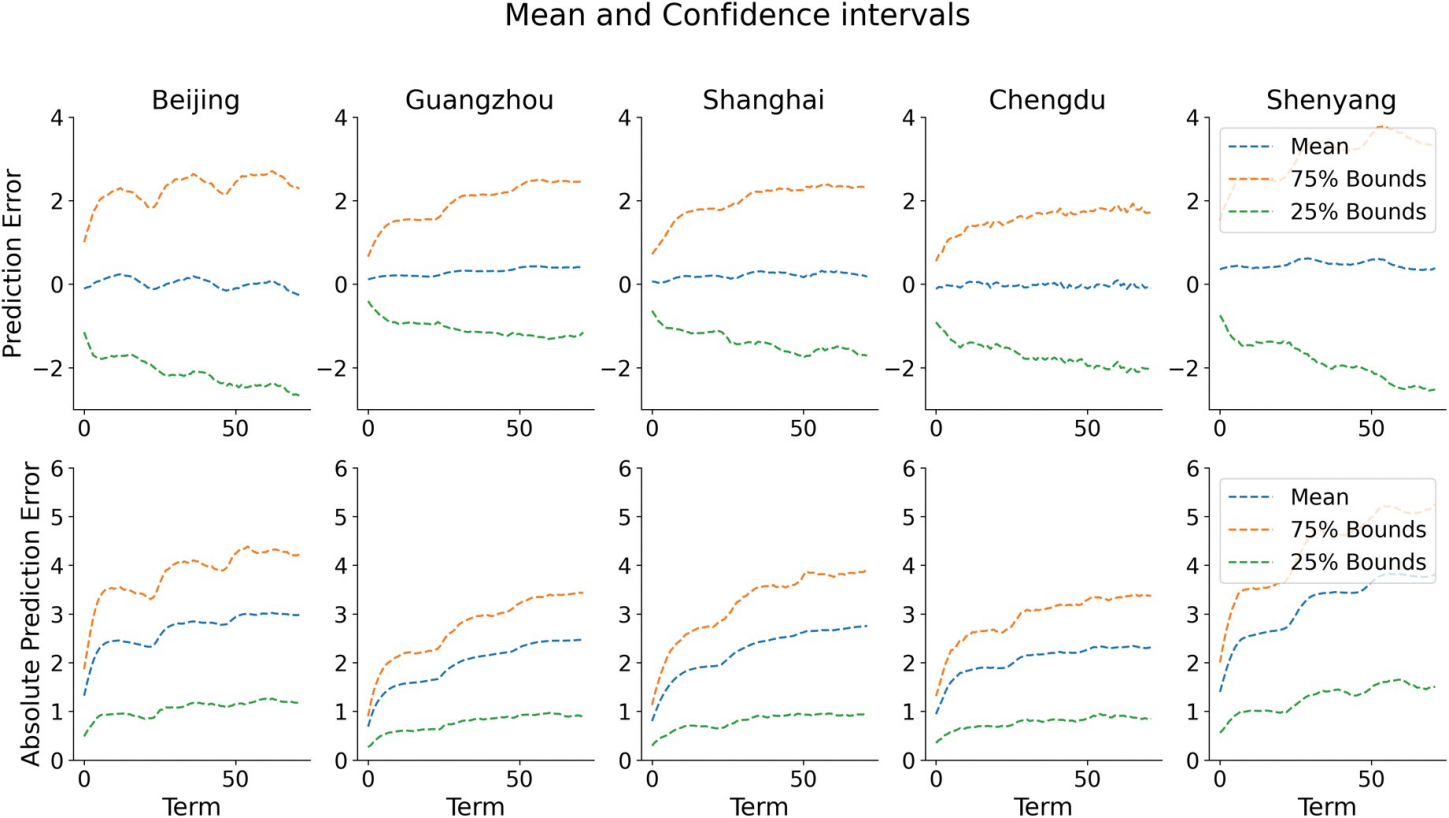
Plot of mean absolute prediction (lower row), mean prediction error (upper row), and confidence bounds (75 percentile and 25 percentile) for the five cities.

The models obtained were used to make a long-term prediction for the next 3 days using information from time *t*−72, the prediction was based on the model in Eq (12). However, the model’s performance was not good for the long-term prediction due to overfitting, we introduced a dropout layer to each of the LSTM layers and a learning rate scheduler, setting a value (0.01) for the decay rate, alpha value (0.6) for the LeakyReLU activation function, and setting a value (0.01) for the L2 regularizer after several experiments to avoid overfitting to the training data. We also optimized the dropout rate for each of the layers using GPyOpt (acquisition function was set to probability index (MPI)). The performance of the models achieved high *R*^2^ values for all the cities (Beijing: 90.39%, Guangzhou: 73.66%, Shanghai: 83.07%, Chengdu: 82.44%, and Shenyang: 88.30%). The predictions obtained these errors (MSE and MAE), i.e. average of the MSE and the MAE, for all the set of 3 days predictions: Beijing (MSE: 10.071; MAE: 2.458), Guangzhou (MSE: 6.717; MAE: 1.925), Shanghai (MSE: 8.789; MAE: 2.171), Chengdu (MSE: 6.253; MAE: 1.920), and Shenyang (MSE: 15.672; MAE: 2.997). Fig 6 presents a residual plot of the actual dataset and the predicted temperature of the last (72^nd^) term using the test dataset. Based on Eq (12), using the test dataset, we obtained predictions for the length of the test dataset for 72 terms. The last prediction term is equivalent to predicting the temperature 3 days later using the current and historical information. We plotted the residual of the actual test set and the last prediction term. Moreover, the error margin increases as the prediction length increases for other sets of predictions compared with the actual dataset. This can be seen in Fig 7 which presents the plot of the mean prediction of the residual (upper row) and the mean absolute error (lower row) with a plot of 50% confidence bound. The confidence interval widens as the prediction term increases which indicates uncertainty in the predictions. The comparison of the test set with the last set of predictions for the test set, we obtained the following errors for each of the cities and the respective *R*^2^: Beijing (MSE: 14.32; MAE: 2.98; *R*^2^: 89.0%), Guangzhou (MSE: 10.55; MAE: 2.47; *R*^2^: 67.61%), Shanghai (MSE: 13.70; MAE: 2.76; *R*^2^: 77.34%), Chengdu (MSE: 8.62; MAE: 2.32; *R*^2^: 81.83%) and Shenyang (MSE: 24.20; MAE: 3.81; *R*^2^: 84.92%).

#### 4.3.1 Comparison of models’ performance

The models’ performance was assessed using the mean squared error, mean absolute error, and the *R*^2^. From Table 1, we observed that the base models, OLS and LASSO, have *R*^2^ values that are less efficient compared to the deep learning models with high *R*^2^ values. The training dataset for the deep learning models performed better than the OLS and LASSO. Similarly, the test dataset for the deep learning models also performed better than the OLS, and the LASSO. The results, therefore, indicate that the deep learning models perform better in predicting atmospheric temperature compared to using the base models (OLS and LASSO). The deep learning models obtained through feature selection and hyper-parameter optimization have proven to be efficient in predicting atmospheric temperature.

## 5. Conclusions

This study presented the use of RNN to predict atmospheric temperature. This was achieved using weather features such as temperature, dew, and wind speed after feature selection. The study focused on predicting atmospheric temperature through the use of Bayesian optimization in selecting the best hyper-parameters for the models. The application of RNN modeling for weather forecasting showed good performance and high prediction accuracies within the range of 72.32% and 100% with minimal errors for the model architecture selected for each city through the use of Bayesian optimization of hyper-parameters and obtained better performance compared to using the base models (OLS and LASSO). The forecasting reliabilities were evaluated by computing the MAE and MSE for the actual values and the predicted values. The performance of the models was also assessed by computing the binary accuracies which estimate how well the predicted values are close to the actual values. Also, the overall performance of the deep learning models when the predicted values and the actual observations were compared obtained a higher *R*^2^ (99.9280%– 99.9779%) than the benchmark models.

In general, the deep learning models obtained have proven to be efficient in predicting atmospheric temperature with minimum errors. The study makes use of five different models, each for one city for the five different cities. There was no single model developed to predict atmospheric temperature for the different cities, the model can be used for different cities but will not perform well compared to when it is used for the selected city. A more complex model architecture would be required to get a model that would be used to make predictions for all five cities.

To apply the backpropagation, we first need to obtain the derivation of the cost function;

$$\frac{\partial C}{\partial x_{n}}=x_{n}-o_{n}$$ (3)

Eq (3) which can also be termed as the loss becomes fundamental for the backpropagation process. Three main equations form the foundation of backpropagation; output layer error (4), the derivative of cost concerning weight (5), and hidden layer error (6).


$$Output\;layer\;error\;\frac{\partial C}{\partial z_{n}}=\frac{\partial C_{n}}{\partial x_{n}}f^{\prime }(z_{n})$$ (4)

$$The\;derivative\;of\;cost‐weight\;error\;\frac{\partial C_{n}}{\partial w_{n}^{ij}}=x_{n-1}^{j}\frac{\partial C}{\partial z_{n}}$$ (5)

$$Hidden\;layer\;error\;\frac{\partial C_{n-1}}{\partial x_{n-1}^{m}}=\sum w_{n}^{im}\frac{\partial C_{n}}{\partial z_{n}}$$ (6)

Eq (6) is the core of backpropagation and after calculating the current layer’s error, the weighted error is passed on to the previous layer. This process continues until the first hidden layer. The weights are updated using the derivative of cost concerning each weight. The value from Eq (5) gives the change in weight in the current layer (*n*).

The updated weight is expressed as;

$${\left(w_{n}^{ij}\right)}_{new}={\left(w_{n}^{ij}\right)}_{old}-\eta \frac{\partial C_{n}}{\partial w_{n}^{ij}}$$ (7)

*η* is the learning rate and decrease during the training process.

To reduce overfitting and improve the model performance, we employ the L2 regularizer regularization techniques. We introduce the L2 regularization technique imposed on the weights within LSTM nodes. The L2 regularizer obtained from the L2 norm presented as ${\left\|w\right\|}_{2}={\left({\left|w_{1}\right|}^{2}+{\left|w_{2}\right|}^{2}+\cdots +{\left|w_{N}\right|}^{2}\right)}^{\frac{1}{2}}$ The loss function and the L2 regularization can be expressed as

$$\mathrm{Loss}=\mathrm{Error}(y,\hat{y})+\lambda \sum_{i=1}^{N}{\left\|w\right\|}_{2}^{2}$$ (8)

where *λ*>0 introducing the L2 regularizer the updated weight is expressed as

$${\left(w_{n}^{ij}\right)}_{new}={\left(w_{n}^{ij}\right)}_{old}-\eta \frac{\partial L}{\partial w_{n}^{ij}}$$ (9)

We then differentiate (8) to obtain a new updated weight equation

$${\left(w_{n}^{ij}\right)}_{new}={\left(w_{n}^{ij}\right)}_{old}-\eta (\frac{\partial C_{n}}{\partial w_{n}^{ij}}+2\lambda w)$$ (10)
